# “Everything is provided free, but they are still hesitant to access healthcare services”: why does the indigenous community in Attapadi, Kerala continue to experience poor access to healthcare?

**DOI:** 10.1186/s12939-020-01216-1

**Published:** 2020-06-26

**Authors:** Mathew Sunil George, Rachel Davey, Itismita Mohanty, Penney Upton

**Affiliations:** Health Research Institute, University of Canberra, ACT, Canberra, 2617 Australia

**Keywords:** Inequity, UHC, Indigenous communities, Access, India, Kerala

## Abstract

**Background:**

Inequity in access to healthcare services is a constant concern. While advances in healthcare have progressed in the last several decades, thereby significantly improving the prevention and treatment of disease, these benefits have not been shared equally. Excluded communities such as Indigenous communities typically face a lack of access to healthcare services that others do not. This study seeks to understand why the indigenous communities in *Attapadi* continue to experience poor access to healthcare in spite of both financial protection and adequate coverage of health services.

**Methods:**

Ethnographic fieldwork was carried out among the various stakeholders living in *Attapadi*. A total of 47 in-depth interviews and 6 focus group discussions were conducted amongst the indigenous community, the healthcare providers and key informants. The data was coded utilising a reflexive and inductive approach leading to the development of the key categories and themes.

**Results:**

The health system provided a comprehensive financial protection package in addition to a host of healthcare facilities for the indigenous communities to avail services. In spite of this, they resisted attempts by the health system to improve their access. The failure to provide culturally respectful care, the discrimination of the community at healthcare facilities, the centralisation of the delivery of services as well as the lack of power on the part of the indigenous community to negotiate with the health system for services that were less disruptive for their lives were identified as the barriers to improving healthcare access. The existing power differentials between the community and the health system stakeholders also ensured that meaningful involvement of the community in the local health system did not occur.

**Conclusion:**

Improving access to health care for indigenous communities would require UHC interventions to be culturally safe, locally relevant and promote active involvement of the community at all stages of the intervention. Continuing structural power imbalances that affect access to resources and prevent meaningful involvement of indigenous communities also need to be addressed.

## Introduction

Advances in healthcare have significantly improved the prevention and treatment of disease globally. However, the benefits of these developments have not been distributed equally across society. In particular, Indigenous populations often have less access to health services than their non-Indigenous contemporaries [1–5]. Even in high income countries non-Indigenous populations experience better health outcomes compared to Indigenous people [1, 3, 6–9] with the additional barrier of poor access to healthcare compounding the situation further [2, 10]. The health of Indigenous communities in India mirrors that of other First Nations’ people across the globe, particularly with regard to life expectancy, maternal and child health and access to services [9, 11]. Acknowledging ongoing gaps in achieving health for all, the World Health Organisation (WHO) proposed Universal Health Coverage (UHC) as a key policy for enabling equitable access to healthcare [12]. Since the 2005 World Health Assembly (WHA), UHC has been adopted globally, with the inclusion of UHC among the Sustainable Development Goals (SDGs) signalling its importance as an international health priority. Despite this nearly half of the world’s population still struggles to access basic healthcare services [13], with Indigenous communities over-represented in this group.

Despite successes in health and development, the South Indian state of Kerala is not immune to the social exclusion and marginalization of Indigenous communities living in the state. Several studies and reports point to greater disadvantage for Indigenous communities, higher levels of morbidity, and poorer access to healthcare in Kerala [14–17]. On typical indicators of population health and wellbeing such as rates of infant mortality, maternal mortality and under five mortality, the Indigenous community in Kerala lags behind others (Table 1) [18, 19].

**Table 1:**
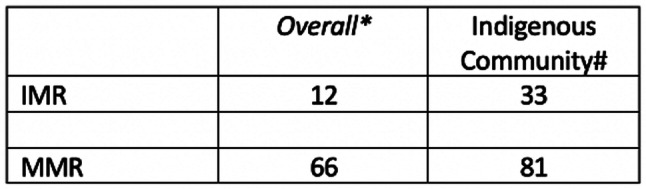
Infant and maternal mortality rates of the indigenous communities in Kerala

Sources: * SRS Bulletin, Sample Registrattion System, Registrar General of India, Vol. 49. No. 1 # Statistical profile of Scheduled Tribes in India. Ministry of Tribal Affairs, Government of India

**Fig. 1:**
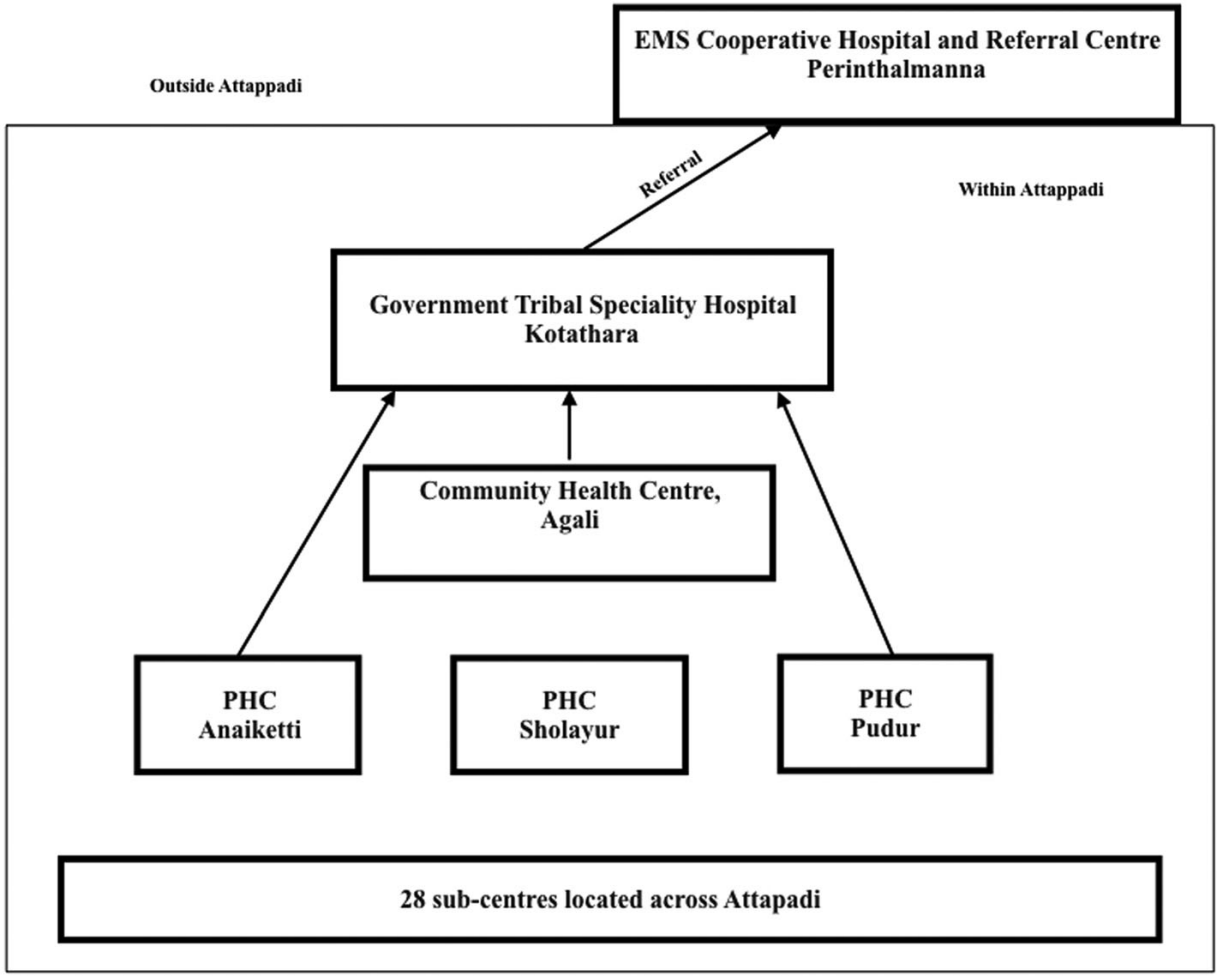
Government Health Facility Network

Attapadi is a tribal^1^ block^2^ located in Mannarghat taluk in the Palakkad district of Kerala, comprising 192 villages inhabited by members of the *muduga*, the *kurumba* and the *irula* Indigenous communities. Healthcare services in Attapadi are primarily publicly provided (Fig. 1) and follow a three-tier system based on population norms as in other parts of India [20]. In addition to services provided at health facilities, each of the PHCs and the CHC have two mobile medical units (MMUs) which consists of a doctor and a nurse each and visits every village periodically. Frontline workers such as Accredited Social Health Activists (ASHAs) and Junior Public Health Nurses (JPHNs) who have responsibility for the villages that are being visited also join the MMU during the visit. In addition to public health system, there are a small number of private healthcare providers which include two private hospitals and a few private clinics.

In 2013, the Kerala Department of Health responded to high levels of infant mortality in Attapadi by implementing reparative measures including upgraded health facilities and the appointment of specialist doctors to improve both the quality of care and the services available. They also introduced a complete financial protection scheme which addressed both direct and indirect healthcare costs ensuring free healthcare services and launched a system to ensure free referral care by specialists [21, 22]. All healthcare services, including diagnostics and drugs, were provided free at the point of service provision and indirect costs such as the cost of travel, loss of wages and costs incurred on other expenses related to accessing healthcare were all reimbursed at fixed rates. In addition to these initiatives, the Scheduled Tribes Development Department (STDD) rolled out specific programmes to promote healthcare services among the community including a financial package to incentivise hospital-based ante-natal care (ANC) and delivery. Acknowledging the importance of social determinants, the Government of Kerala also established an active review mechanism to ensure inter-sectoral collaboration and promote convergence among the various departments (Table 2).

**Table 2:** Interventions to improve universal access to healthcare for Indigenous communities in Attapadi

1. Complete financial protection for direct costs of all treatments including tertiary and specialist referral care	
2. Upgrading of health facilities in Attapadi including the tribal speciality hospital and appointment of doctors including specialists and other healthcare personnel across the various health facilities in Attapadi	
3. Addressing indirect costs for accessing healthcare through reimbursement of travel costs, providing free food for patient and one family member during hospitalisation, reimbursement of loss of wages at a fixed daily rate for one family member who remains with the patient during hospitalisation	
4. Special salary package for healthcare workers opting to work in Attapadi	
5. Establishment of a special referral arrangement with EMS Cooperative Hospital and Research Centre for tertiary care	
6. Establishment of mobile medical units with dedicated teams under each of the three PHCs and the CHC in Attapadi to provide screening, limited primary treatment and immunisation services to those living in remote villages	
7. Establishing a formal mechanism chaired by the nodal office of the state government to review the work of multiple government departments on a monthly basis and promote inter-sectoral coordination between various departments to address the challenges faced by the Indigenous communities living in Attapadi	

Yet despite these measures, the Indigenous communities in Attapadi continue to experience high levels of infant mortality and poor health [23–25]. We therefore undertook a qualitative exploration of the socio-cultural factors affecting healthcare access in Attapadi to increase our understanding about why these health disparities are ongoing, so as to be able to identify practical solutions.

## Methods

### Initial conceptual framework

We used the conceptual model of healthcare access described by Levesque and colleagues as our starting point [26]. In this model access is the opportunity to identify healthcare needs, to seek healthcare services, to reach, to obtain or use health care services and to have the need for services fulfilled. Access results from the interaction of the abilities of an individual or the population seeking access and the attributes of the health system providing the services. The population must be able to perceive, seek, reach, pay and engage with the service. The corresponding health system attributes are approachability, acceptability, availability and accommodation, affordability and appropriateness. These abilities and attributes are not completely independent, and they often influence each other.

While this conceptual model guided our initial definition of the aspects of access that we planned to explore (Table 3), it became apparent in our early interviews that structural injustice and the lack action on social determinants of health were major issues not explicitly covered by this model. In keeping with the emergent nature of qualitative work, we explored these topics in subsequent interviews in order to understand this further, thereby allowing our fieldwork and analysis to be grounded in the data that emerged.

**Table 3:** Initial themes explored during interviews and FGDs

Community	Healthcare providers
• Experience of managing ill health in the family/for self • Perception of needs and healthcare requirement • Initial management of healthcare (if any) • Previous arrangements including the use of alternative systems • Experience of seeking formal healthcare • Experience at health facilities • Facilitators for seeking healthcare • Challenges faced in obtaining care • Experience after obtaining care/follow up	• Opinion on healthcare facilities/ services being provided • Details on the organisation of health care services • Facilitators of healthcare provision • Challenges faced in providing care • Strategies to improve access to healthcare • Opinion on the role of alternative systems in enabling healthcare access

### Data collection

Ethnographic fieldwork was conducted between August 2018 and January 2019 and again between August 2019 and October 2019 in Kerala by the first author (MSG) who is fluent in the native language of the participants (Malayalam/Tamil) and has prior experience working with marginalised Indian communities. Prior to commencing data collection, MSG lived among the Indigenous population for 6 months adopting their customs and building rapport with the community. This enabled understanding of context and local traditions including protocols for outsider interactions with the community. Two visits were made to each fieldwork site (villages). The first was to meet the village chief, explain the study, its purpose, and what was required should the community wish to participate. Data collection was carried out in the second visit once permission was granted by the village chief. This approach built trust and rapport between MSG and the participants before data collection, facilitating a frank exploration of issues.

In-depth interviews (IDIs) with the community took place in their homes, whereas focus group discussions (FGDs) took place in the common courtyard in the village which was used socially. IDIs with health system participants and key informants (K.I.s) took place at a location in their homes or offices depending on their preference. Participant observation (PO) involved being present in the common areas of the health facilities during the working hours and observing interactions between the community and the healthcare providers and between the various healthcare personnel working at the facility. This allowed us to observe and record the dynamics of the interaction between the various stakeholders. PO was also carried out in the villages and helped to document health traditions, cultural practices and the challenges of accessing a health facility, particularly for those living in remote villages. MSG also travelled with the mobile medical units (MMUs) in order to observe the services that they provided to remote villages in Attapadi. MSG visited two Indigenous health projects in south India where he was able to conduct participant observation of their activities as well as interact with health service providers.

#### Sampling

Participants were identified through theoretical sampling, with initial interviews providing new topics that were explored in subsequent interviews [27]. In each of the villages, information rich participants were sought which included those who had experience of seeking healthcare and also those who had resisted attempts by the community health worker or others from the health system to seek treatment. Twenty-four in-depth interviews (IDIs) and six focus group discussions (FGDs) were undertaken with three different Indigenous communities living in Attapadi. A further 17 IDIs were conducted with local healthcare providers. Six Key informants (KIs) including academics, and experts on UHC and tribal health in south India were also interviewed (Table 4). Data collection continued until saturation of themes was reached. Fifty-two instances of participant observation were conducted at the different villages, the health facilities in Attapadi and the hospitals at the two tribal health projects outside Attapadi. Detailed field notes were recorded and integrated into the analysis.

**Table 4:** Sampling framework

	*Irula*	Muduga	Kurumba	Total
Indigenous Community	IDI: 9, FGD:1	IDI: 7, FGD: 1	IDI: 8, FGD: 4	IDIs: 24, FGD: 6
Healthcare Providers	Doctors	CHWs^a^	Others^b^	
	8	6	3	IDIs: 17
Key Informants	Academia	Indigenous health experts		
	2	4		IDIs: 6
Participant Observation	Community	Health Facilities	Tribal Health Projects^c^	
	24	26	2	52 units

^1^CHWs involved both Indigenous and non-Indigenous frontline healthcare workers working in the government health system in Attapadi

^2^Staff working at the various health facilities other than doctors, nurses or CHWs

^3^Refers to participation observation carried out at the health facilities of two tribal health projects that were visited outside Attapadi

#### Data analysis

With the consent of participants interviews and FGDs were audio-recorded, transcribed, translated into English and cross-checked against the original recordings. The translated transcripts were coded using a reflexive and inductive approach to allow themes to emerge from the data. Once the initial open coding was complete, axial coding was used to express the relationships between the various themes as they arose from the data. A second author (PMU) independently coded a sub-sample of the transcripts and the two sets of analysis were compared. This approach to analysis was taken in order to let the data drive our categories and themes rather than using a deductive or a quasi-deductive approach of fitting our findings to a particular framework. Any discrepancies in coding were reviewed and resolved by in-depth discussion and negotiated consensus. Coding of transcripts was carried out using the software package Atlast.ti 8.4.2.

#### Establishing trustworthiness

Multiple strategies were adopted to ensure that our findings were rigorous and reflected the lived reality of the community for accessing healthcare. Strategies including multiple forms of member checking [28–30] and triangulation [31] were used for validating the data. Synthesized findings from the initial analysis were discussed with all participants with the opportunity provided for them to comment and add new data if needed. Feedback received during the member checking process were integrated into the findings that are presented in this paper. Triangulation of the findings is a well-established method in qualitative research to develop a comprehensive understanding of what is being studied and enhance the quality and credibility of qualitative analysis [31]. Data source triangulation [32] was carried out by comparing the perspectives of different stakeholders on the key findings. Methodological triangulation [32] was carried out by comparing the data that was generated across IDI, FGDs, and participant observation. Finally a report on the functioning of the local health system in Attapadi commissioned by the STDD was also used to triangulate the key findings presented in this paper [33].

#### Ethics approvals

Informed consent was gained from individual participants prior to data collection. The Human Research Ethics Committees of the University of Canberra (20180074) and the Indian Institute of Public Health Delhi (IIPHD_IEC_03_2018) provided ethical approval. Regulatory permissions were obtained from the Kerala Department of Health (GO(Rt)No2677/2018/H&FWD), as well as the local administration in Attapadi.

## Results

While Attapadi has three different Indigenous communities, we did not find major differences between the three communities regarding what mediated access to healthcare in Attapadi. While presenting our results we report the major themes that evolved with respect to healthcare access and present the perspectives of the different stakeholders who participated in our study in each of the themes.

### Marginalisation of indigenous culture and healing traditions

The understanding of healing and disease in most formal health systems follows a biomedical framework where health and illness are defined primarily from a clinical perspective. While this dominant model has come to characterise most health systems across the world, the significant role played by culture and beliefs regarding healing and causation of ill health especially among Indigenous communities is well known [34, 35]. The Indigenous communities in Attapadi took a holistic approach to life and health which was rooted firmly in their cultural tradition. While discussing healing and health, Indigenous participants would often link the environment, their food and their connection to their ancestors as essential for good health.

While Indigenous communities across the world have their own system of traditional medicine, the actual practise of this system is considered the preserve of a few healers or *shamans* [36]. However, we discovered that a key aspect of the Indigenous healing traditions in Attapadi was that it was not restricted to a few healers. Community participants explained that everyone in the village was expected to know the different medicinal plants and their usage. Yet another example that emerged in our interviews was the Indigenous practise of birthing at home. The husband was responsible for assisting at the birth of his child instead of depending on anyone else either within the family or from the village. This knowledge was passed on from father to son and was considered an essential knowledge when a young man got married. Only in case of complications during childbirth would the husband find someone else from the village to assist him.

However, several participants described how traditional healing had been superseded by the modern health system. The community felt that outsiders, in particular the health department staff, not only ridiculed their practices as ineffective superstition, but also strongly discouraged their use. This eventually has led to the decline in the use of traditional medicines among the Indigenous communities. They also described how their younger generation were not interested in learning about these traditional medicines. Some of the community participants felt this decline in use of their traditional medicines deprived them of first-line remedies that their communities had practised for various ailments. While the local health system felt such remedies hampered the provision of “good quality” healthcare, communities that lived in remote locations explained that the knowledge and use of traditional medicines were an important part of their lives and not using them rendered them more vulnerable.

“If we use any of our traditional medicines and anyone in the health system comes to know about it then they will scold us for it. But when we are living so far away from the health facilities, we need to use our medicines. At least as a home remedy, at least as a first line treatment.”

Indigenous community, IDI, TK 2

“Our healers they used to treat so many diseases. In those days our ancestors had to go and hunt in the forest for food, and what do you think they would do if they were bitten by a snake? They had to find ways of healing themselves. There were no hospitals like we have now. Now very few people use any of our herbs. In fact, many of us don’t know about it.”

Indigenous community IDI, TI 13

Health system participants were generally unaware of local cultural beliefs about healing and did not consider this to be relevant to the services they provided. The general understanding was that these practices were superstitious at best and harmful at worst. Beliefs and practices around the significance of birthing on country, death etc. were considered as impediments to the delivery of effective healthcare services. There was no acknowledgement of the need to integrate local beliefs into the health system’s practice so as to provide culturally safe and respectful services. One of the observations during fieldwork was how husbands of pregnant women from the Indigenous community would not cut their hair till the child was born and named. Some of the doctors pointed this out as an example of a lack of hygiene among the Indigenous community, noting that they continued this practice despite being told it was unhygienic. In contrast community participants explained that this was part of their culture and an expression of the husband’s affection towards his pregnant wife. Yet another example was the fear of being referred to a hospital outside Attapadi, especially among the elderly. It was common to find the elderly refusing to access healthcare and remain in their villages despite efforts to convince them to seek care. This was reported as a barrier to providing good quality care by the healthcare professionals. Two major reasons for this behaviour described to us included the commonly held belief among the elderly that once they left the hills of Attapadi, they were no longer under the protection of their ancestors. Secondly, the community also observed that many of those who had been referred to places outside of Attapadi in the past did not survive. This might have been due to the fact that most of those referred were already in very poor health to begin with. However, it left a negative impression among the community and reinforced their beliefs that it is not safe for them to leave the hills of Attapadi to seek treatment. Doctors referred to such beliefs as an yet another example of superstition which had to be rooted out, rather than trying to understand the perspective of the community. This failure to understand and respect the important role played by culture in the lives of the Indigenous led to situations where the community resisted well-meaning advice offered by healthcare providers.

Quite contrary to the approach at Attapadi, we found that successful Indigenous health projects in India proactively tried to understand and integrate culture as a part of the approach to service delivery. One of the key informants who worked for a very well-known Indigenous health project in another part of south India pointed out that adapting healthcare delivery to Indigenous culture was important.

“Culture is very important when we deal with Indigenous communities. For example, the *betta-kurumbas* have a practise where, when a woman goes into labour all the women from the village gather around the house. It is like a way of showing solidarity with the girl who is about to deliver. When we realised this, we had negotiations with the community and agreed to let their women gather in the meeting room and not outside the labour room. As a result, we were able to get more people to come to the hospital to deliver and they were also happy that we took into respected their traditions.”

Key Informant, IDI, KI 5

Such openness to Indigenous culture and traditions and the ability to negotiate and arrive at approaches that was culturally sensitive while being clinically acceptable was grossly lacking in the local health system at Attapadi.

### Lack of community engagement

Several village chiefs pointed out that no one ever asked for their opinion regarding healthcare provision for their community. They felt that given their ‘illiterate’ status, doctors and other health professionals did not see their views as important. Healthcare staff did not inform village chiefs even when an MMU visited a village. They failed to use this as an opportunity to actively engage the community. MMUs would generally arrive at the villages after most of the community had left for their daily work. Village chiefs who were interviewed pointed out that if they knew details about the MMU’s visit to their villages in advance, they could discuss it with the community and convince them to stay back in the village for that day. The exclusion of village chiefs and their councils - the traditional decision makers, was common across all programmes. Furthermore, a senior staff member from one of the health facilities revealed that while there was a hospital management committee with Indigenous representation, it was more focussed on the development of the hospital than on improving community engagement. This approach contrasted with the custom among the Indigenous communities where all common issues had to be discussed in village meetings and decisions arrived at by the community.

“Even though I am the village chief (*moopan),* nobody has asked me anything so far. Even the doctors who come here on medical camp they don’t ask me for my opinion. They do things as they think is best. Our opinion is not taken.”

Indigenous community FGD, TK 12

This lack of community engagement lead to initiatives with no value either to the community or the health system. Such initiatives gave the impression to outsiders and higher officials that efforts to promote community engagement existed, but in reality, these were nothing more than symbolic gestures undertaken as tick-box exercises. Another example was the feedback system provided at the tribal speciality hospital. None of the participants who had received hospital services knew about its existence. Furthermore, the feedback form was in English and Malayalam two languages that most tribals do not read. One of the doctors interviewed acknowledged that the form was more for the record of the external evaluation team that assessed the hospital. Lack of adequate engagement was also reported by individual participants who had received healthcare services. One of the common issues raised in the interviews was how doctors did not spend adequate time examining patients or discuss their prognosis in detail with them.

“I was with my brother when he was being treated. They did not tell me why he was having this problem. They just told me I will have to take him to the hospital in Thrissur for treatment. Other than that, they did not tell me anything about it.”

Indigenous community, IDI, TM7

The exclusion of the community from decision making processes related to the health system added to a lack of belonging that the Indigenous communities felt about the health system. A key informant who headed a well renowned tribal healthcare initiative in south India pointed out that unless there was strong community ownership, interventions carried out among the Indigenous were bound to fail.

“Here the golden rule is that everything has to be discussed with the community first; we have to take them along. It is not easy; it slows things in the beginning. But unless the community is on board, our work is not going to succeed in the long run.”

Key Informant, IDI, KI 3

### Centralisation of healthcare services

A network of healthcare institutions with trained staff and appropriate infrastructure was present in Attapadi (Fig. 1). In spite of this, most healthcare services including ANC were provided only at the tribal speciality hospital. The general trend among healthcare professionals at the Primary Health Centres (PHC) and Community Health Centre (CHC) was to refer patients to the tribal speciality hospital. When asked why they did not treat them at their facilities, doctors explained that this was because the tribal hospital had specialists and better facilities. In contrast, community members described being afraid to go to big hospitals away from their homes. Many described feeling disoriented in the tribal hospital, a large building with several rooms and offices. Even more worrisome was the fear that they would be referred outside Attapadi for treatment to a bigger hospital. Some of the participants shared stories of how they had travelled for several hours to reach the hospital, even though there were other health facilities closer to their villages.

“If someone falls sick in those villages, we are expecting them to come to the hospital which is so difficult to reach.”

Healthcare Provider IDI, TP1

“Here in the PHC they won’t do anything, for anything we have to go to the tribal hospital at Kottathara only.”

Indigenous community FGD, TI 10

A fallout of the trend to centralise healthcare services in Attapadi, was how other facilities such as sub-centres, PHCs and the CHC were being neglected despite possessing excellent infrastructure and adequate healthcare personnel at these centres. Additionally, this also led to overcrowding at the tribal speciality hospital compromising the time available per patient for diagnosis and treatment thereby affecting the quality of care delivered.

### Forced compliance

In efforts to improve community access, the local health system tried to enforce compliance with its programmes and initiatives. Pregnant women were required to make monthly visits to the tribal hospital in Kottathara to receive ANC care. This put many pregnant women, especially those who lived far away, into great difficulty. The community did comply with the requirements of the health system, but primarily from fear of the negative consequences of non-compliance. They felt that the health system was unable to appreciate the context of their daily lives implementing interventions that were disruptive. Healthcare providers stated that they acted as they did for the community’s own good. Forced compliance also had a negative impact on the work of community health workers who explained that such incidents made their work difficult as they lost community trust.

“Now if they know there is any pregnant woman here then they will keep a note of it and before the time comes, they will come and take them away. Even if you go and hide in the forest they will come and take you to the hospital. Even one month before your date they will take you away even if you are not happy with it.”

Indigenous community IDI, TI8

Closely linked to this enforced compliance, was the fear expressed by the community about receiving inpatient care at the hospital. Doctors found it difficult to communicate effectively with their patients and the community resisted some of the efforts of well-meaning doctors to provide certain services for them. The lack of trust among patients was discussed by some of the doctors who felt that their experience in Attapadi clearly exposed a gap in trust between the healthcare providers and the community.

“The sight of the labour room makes them worried; they get very scared; I have seen that”

Medical officer IDI, MO 1

“Now I won't go, I am alone, it is scary to go alone and stay in the hospital.”

Indigenous community IDI, TI 8

“They will never cross a certain line and get close. The personal touch and trust that should characterise a doctor patient relationship, I find it is missing here completely.”

Medical Officer IDI, MO3

Fear was also expressed by the community about being referred out of Attapadi for any treatment.

The topic of fear was also brought up by health system participants in the context of working in Attapadi. Doctors in particular pointed out that negative media coverage that followed incidents such as infant or maternal deaths, made them very cautious about treating cases especially related to maternal or child health. Doctors were of the opinion that if something went wrong, they would have to face the consequences and higher officials would not back them up. Hence, they referred complex cases to another centre even if it was located outside Attapadi so that they did not face any trouble in case something went wrong.

### Stigma and discrimination

Most participants described feeling discriminated against by the health system. This was reported by community members who had received health services, and by Indigenous staff members in the health facilities. Community participants described the condescending manner in which non-Indigenous staff engaged with them at the health facilities. Indigenous healthcare workers also noted that they were treated differently by the non-Indigenous staff. One participant who had resigned from her job because of this, explained that it was distressing to be constantly seen as different. Some participants felt that the image of a tribal as an “uncivilised savage” still persisted. Even though such attitudes were not stated explicitly, the community was unanimous that they were stigmatised by the dominant community, including the health system. It was significant that not a single Indigenous participant, expressed a sense of belonging and ownership about the health system.

“I left because they used to see us tribal staff differently from the others. I did not like it, so I quit my job.”

Indigenous community IDI, TM8

“Never once did I feel this is “our hospital”. The reason is because they never see us as part of them. And neither have I felt that. They differentiate.”

Indigenous community IDI, TK 4

Most of the non-Indigenous participants from the health system were not willing to accept that there was stigma or discrimination against the Indigenous community. They felt that everything was provided for the Indigenous communities and they did their best. Indigenous community members also agreed that direct acts of discrimination were rare, but they could sense unconscious bias which took shape and form in some of the language that was used by healthcare providers.

### Addressing the broader determinants of health

One of the key themes that emerged from the community was how the loss of their lands and restricted access to the forests impacted their ability to have a nutritious diet. The loss of lands and the resulting marginalisation of the Indigenous community in Attapadi has been long acknowledged by several governments in Kerala. As a result, many of the Indigenous community members are unable to grow their own food which was the custom in the past. Furthermore, with forest laws that in effect prevented communities from hunting small animals for food- a practise that they were used to for generations or go into the forest to collect roots and other produce freely, access to several sources of food was curtailed. Older community members spoke of the special diets provided in the past to pregnant women. Specific wild root vegetables were eaten to improve the health of the mother and their unborn child. However, access to these nutritious diets has declined over the last four decades. Despite several processes set in motion by various governments, most of the lands have not been restored to the Indigenous community, depriving them of the chance to cultivate their own food as in the past. As noted by one of the doctors, it is unrealistic to leave aside socio-cultural determinants of health and expect clinical solutions alone to address community health issues.

“Whenever people talk of health, I ask them about our land. Unless we eat nutritious food, how can we be healthy, what is the use of these big hospitals?”

Key Informant IDI, KI4

“Let’s say for example you are bringing in a 20-year-old girl for delivery and I have seen girls who are secundigravida at that age. Even if it is a primigravida the weight of the girl will be like 37 kgs. Now you tell me, how can I in a short time make her deliver a baby that is above 2.5 kgs.? For that to happen I should be sitting here and doing some magic. That is why I say you can’t just do these things sitting here in the hospital.”

Medical officer IDI, MO3

Loss of lands not only impacted upon the ability to have a nutritious diet but also led to the loss of livelihoods for those who were engaged in the cultivation of millets and other traditional crops on their land. Moreover, during fieldwork, several Indigenous village chiefs took MSG around and pointed out the several instances where cultivable lands closest to the main sources of water in Attapadi no longer belonged to the Indigenous people. Land was not merely a physical asset for the Indigenous people but had a far deeper cultural and spiritual significance in the form of their connect with their ancestors and their traditions and rituals. The inability to regain what was lost was an issue of concern for Indigenous participants and their elders.

“I have been asked multiple times by officials if I can relocate my village from where it is now to a place closer to other facilities including hospitals. But how can I leave this place? Our ancestors are buried here, and our belief is that they live here in this environment. Others do not understand this. Only an Indigenous person will understand why our land is so important for our way of life.”

Indigenous community IDI, TK4

Indigenous participants pointed out that while the government was upgrading health facilities and instituting other measures around healthcare, they were not taking adequate action to restore lands and address underlying determinants of health which were important to them. This lack of progress on the social determinants of health, meant that structural inequities continued to be a feature of the Indigenous communities living in Attapadi.

### Financial protection alone did not improve access

All healthcare services including referrals to tertiary institutions outside Attapadi were free for the Indigenous community after 2014. In addition to direct costs, indirect costs such as those for transport, food, medicines, loss of wages for an accompanying carer, were all addressed by a special renumeration package. The STDD also implemented schemes to ensure that indirect costs incurred from accessing healthcare services were reimbursed. Additionally, financial incentives were offered in order to promote ANC and hospital deliveries. All participants were aware of the renumeration available, and that they did not have to pay for any care received. Yet access to healthcare was not universal.

“No, it is not a burden. In fact, we used to pay for the transport and all. For ANC too we are paying. For attenders we pay, we give them free food when they are here. We pay something that is the equivalent of a day’s wages.”

Medical officer IDI, MO1

“Everything was free of cost we did not pay anything for his treatment. They gave us food there itself and also for the attender they gave 100 rupees per day.”

Indigenous community IDI, TM7

Key informants described the current approaches and debates around provision of UHC in India as inadequate, having narrowed their focus to the provision of financial protection in the form of insurance packages linked to clinical services, provided through a network of public and private hospitals. This they felt would not be adequate to ensure truly universal healthcare access, however in the face of a global initiative such as UHC, there was a lot of pressure to implement an insurance-based financing mechanism as the way forward. All KIs emphasized the need for decentralising of both planning and the process of conceptualizing the barriers to access and solutions that needed to address them. One of the errors of global initiatives such as UHC was to universalise one intervention and expect diverse populations to respond to it in the same manner.

“I do think that the present approach to UHC is flawed as it is mostly a focused-on hospital-based care.”

Key informant IDI, KI 1

“What we need to do is to universalise access, for this we cannot universalise one intervention as the context in Attapadi differs from the context in a big city. So how can you say that one broad approach will ensure access for all?”

Key informant IDI, KI 2

“You cannot import something from outside and expect it to work with an indigenous community”

Key informant IDI, KI 3

## Discussion

Access to healthcare is a multifaceted concept and has various determinants on both the demand and supply side. Levesque et al. expressed this in the form of a set of abilities and attributes that influence access right from the perception of healthcare needs up to the utilisation and consequences of accessing healthcare [37]. Our study identifies that the mere presence of health facilities and complete financial protection provided by the government had limited success in addressing the healthcare needs of the Indigenous communities in Attapadi. The present approach while providing free healthcare, diminished the various abilities of the Indigenous communities to access healthcare and weakened the attributes of the local health system (Table 5). A key issue to emerge which may explain this finding, was that while the healthcare provided was technically and clinically sound, it did not acknowledge the cultural and traditional values of the Indigenous community. The health beliefs of Indigenous communities are rooted in unique cultural values, obligations and ancient traditions [38–40]. Indigenous health traditions typically understand health in a holistic manner and include respect for land, kinship structures as well as connect with ancestors as important aspects of healing and wellbeing [41, 42]. The provision of culturally safe care which both respects and reflects these values is an important facilitator of better healthcare access, that will ultimately lead to improved health outcomes for Indigenous communities [43–45]. The lack of efforts to integrate traditional beliefs around healing, death and connection to ancestors into the health system’s approach to providing services, led to passive resistance from the communities. Further, the stigmatisation of traditional medicines as mere superstitions meant that the communities were deprived of home remedies which were traditionally used as first line treatments. It is pertinent to note here that such exclusion of Indigenous values and traditions was not restricted to the health system alone but rather reflected the majority view in society about Indigenous people. One approach to improving the well-being of excluded communities such as the Indigenous tribes, has been for the state to locate the cause of their lack of development in the sociocultural aspects of their lives [46]. Under this approach, Indigenous communities can only gain better access to services such as education, healthcare etc., by adopting the ideas and values of the dominant culture. Unfortunately, this approach does not acknowledge the possibility that the lack of access to services may actually result from governance processes dominated by the values of the prevailing culture [46]. Such majoritarian approaches lead to well-meaning interventions, which rather than being the medium of inclusion, reinforce exclusionary processes.

**Table 5:** Major findings and their impact on abilities and attributes of framework to access by Levesque et al

Findings	Community	Health System	Impact
Marginalisation of culture and traditions	• Deprived them of use of first line home remedies • Beliefs around birthing on country, death rituals etc. had impact on access to healthcare	• Dismissed culture and traditions as mere superstitions • Was unsure of how to handle or integrate it into healthcare delivery	Abilities • To perceive, to seek Attributes • Acceptability
Lack of community involvement	• None of the village chiefs consulted on service delivery mechanisms • In stark contrast to traditional decision-making mechanisms in the community	• Consultation was more symbolic • Involvement of village chiefs and elders more to ensure compliance to programmes	Abilities • To perceive, engage Attributes • Approachability, engagement
Centralisation of healthcare services	• Led to spatial exclusion and isolation of community from facilities • Delayed care seeking • Compromised quality of care	• Promoted centralisation on the premise of providing better care	Abilities • reach Attributes • Availability
Forced compliance	• Resented forced compliance to programmes and directives • Lead to fear and lack of trust in the health system	• Pointed out this was used as last resort for the benefit of the community	Abilities • To engage, to seek Attributes • Acceptability
Stigma and Discrimination	• Reported universally by Indigenous community • Larger approach by everyone including health system • Unconscious bias which was picked up by community • Led to lack of ownership about health system and impacted trust	• Non-Indigenous health personnel denied any stigmatising attitudes or practise • Indigenous healthcare providers confirmed that differential treatment and attitudes were a reality	Abilities • To seek, to engage Attributes • Acceptability
Addressing the broader determinants of health	• Raised the importance of land and access to larger social determinants	• Some awareness of the impact but pointed out most of the action required was out of the mandate of the health system	Not addressed by Levesque et al. but emerged as important in the context of the Indigenous communities.
Financial protection	• Aware of free healthcare including referrals • Aware of reimbursements of indirect expenses and other schemes	• Implemented a complete financial protection package to take care of both direct and indirect costs	Abilities • To pay Attributes • Affordability

Culturally safe care ensures that traditional healing practices are integrated into the local health system, allowing care that is delivered to engender respect for the customs and culture of the Indigenous community. Cultural safety also requires the health system to engage proactively with local communities, obtaining their collaboration and involvement where possible [47]. Our findings suggest that community engagement was superficial and the power differentials between health system stakeholders and the community was a key barrier. Culturally safe healthcare impacts the ability to both perceive and seek healthcare, whilst also enhancing the acceptability and approachability of the health system among the community.

Discrimination against ethnic groups and Indigenous communities is a well-documented issue, shown to have serious repercussions for the health of these communities and their ability to engage with non-Indigenous led systems [38, 48–51]. We found discrimination by healthcare providers against the Indigenous communities in Attapadi was prevalent, mirroring the wider societal view that these are broken communities that need to be fixed. This discrimination emerged as unconscious bias [50], reflected in the condescending attitudes and language used by healthcare professionals when speaking to or about the community, their dismissal of Indigenous health traditions and the manner in which the health system placed the responsibility for poor health outcomes on Indigenous community beliefs and actions. These entrenched attitudes are an important barrier to meaningful Indigenous engagement with the health system and its representatives. Such attitudes clearly had an impact on the ability of communities to engage with the healthcare providers. Some of the healthcare providers we spoke to acknowledged the lack of engagement from the community, but failed to realise that this should be a two way process, and that the communities also felt they were not engaged appropriately by the local health system.

A key observation of this study was of the centralisation of healthcare delivery. This was reported as a significant barrier by the community who not only expressed their discomfort and fear of staying in a large hospital, but also highlighted their hardship in travelling the long distances to reach the hospital. The centralised approach to delivering healthcare from within a large institution meant that interaction between most of the healthcare providers and the community was carried out within the hierarchical set up of the hospital. This also increased the social distance between the community and the health system personnel who were seen as the others. Decreasing the social distance between providers and users of health services has been shown to have positive benefits [50]. While designing UHC interventions, policy makers and programme managers need to ensure that their services are not just clinically sound, and culturally safe, but also geographically accessible to the community. A decentralised approach to the provision of healthcare services with emphasis on providing services as close to the community as possible would make health services more available and adaptable to the community.

The quality of healthcare services provided is an important component of UHC [52]. Multiple definitions and frameworks try to unpack what is meant by quality of healthcare [53, 54]. How we define quality however depends on the components we choose to assess [55]. In defining the care that was delivered for the Indigenous people in Attapadi, the local health system focussed on technical and clinical protocols and quality. However, a key component of quality recommended by the WHO and several others is providing care that takes into account the aspirations and culture of the local community [54]. Failure to do this as has been shown in the literature to lead to a situation where the users expectations are not addressed [56]. This was the case in Attapadi and even those who had received healthcare services expressed dissatisfaction with their experience with the process.

Despite an acknowledgement of the importance of social determinants of healthcare access, an understanding of how power or the lack of it shapes access to resources including healthcare is lacking [57–59]. As described by the WHO charter for health promotion, reducing equity of access to healthcare requires empowering marginalised communities to negotiate healthcare that is sensitive, safe and acceptable to them [60]. The lack of this enablement meant that many of our Indigenous participants choose to resist attempts to force them to comply with the requirements of the health system or choose not to seek care when they needed it. While the framework of access developed by Levesque et al. talks about the importance of culture as an attribute of the care that is provided by a health system, and social factors and empowerment of communities as important abilities of individuals and communities seeking care, its focus is on the processes involved in access to health services and thus it does not go beyond the health system. Evidence has however shown that responsive health systems must take into account other aspects of the society in which they are situated if we are to address health inequalities (Greenhalgh 2018). Thus we must move beyond the notion of a health system which is divorced from the rest of the broader sociocultural and political system and engage with uncomfortable issues such as structural inequity whereby government policies - or inactions - keep some groups from obtaining the resources they need to better their lives. Recognition of how groups become marginalised in the first place and addressing these inequities should be seen as integral to improving access to health care, not as a separate topic. As noted by Greenhalgh, structural violence explains how the opportunities and abilities of some groups are hindered, resulting in an unfair distribution of the burden of disease, and moreover why health systems ‘still so often exacerbate, rather than ameliorate, these vulnerabilities’ [61].

Our findings highlight the importance of social structures for Indigenous communities and the adverse impacts of structural inequities on their health and ability to access healthcare. The lack of timebound action on the social determinants, especially returning their lands, gave the impression to the Indigenous communities that they had to continue in their status quo while depending on handouts from the government while key structural issues were bypassed. Addressing social determinants of health such as restoring lands, access to natural resources and so on are key to empowering Indigenous communities, restoring trust in the system and so improving access to healthcare [62]. The failure to acknowledge the structural inequity in which these communities live means we reduce their health and the determinants of access to a series of clinical issues that need corresponding interventions to resolve them. This approach has been shown to reinforce and perpetuate prejudice as well as reinforce feelings of low self-esteem and disempower Indigenous communities in other contexts [63, 64].

While UHC as a concept is diverse, most interventions to promote UHC are implemented as financial protection schemes that address costs related to accessing healthcare [52]. Indeed, most debates around UHC limit themselves to service provision and financial protection. This study provides evidence that in the context of Indigenous communities, this approach to UHC is ineffective. Hence it is essential for UHC interventions to acknowledge the vital role that local socio-cultural context plays in healthcare access. If interventions to promote UHC are to reach marginalised groups such as Indigenous communities, they must be culturally safe, locally relevant and planned with active involvement of the community. For this, several areas including approaches to policy and programme (Table 6) need to be revisited and revised to ensure culturally safe health care practices are implemented to improve UHC.

**Table 6:** Recommendations for improving access to healthcare for the Indigenous community in Attapadi

Recommendations to improve access to healthcare for the Indigenous	
**Policy**	
1. Decentralise services to ensure that appropriate services are delivered at each of the sub-centres, PHCs, and CHC with the Speciality Hospital acting more as a referral hospital for secondary and specialist care.	
2. Training and sensitisation of medical officers and other health staff an essential part of working among Indigenous communities	
3. Address social determinants of health including return lands that rightfully belong to the Indigenous communities in Attapadi	
4. Appoint local Indigenous youngsters as community health workers to work in Indigenous villages	
**Programme**	
1. Form health committees in villages involving the community health worker, the village chief or a representative and engage them in decision making about local health programmes.	
2. Form council of village chiefs from all three communities with advisory role to guide delivery and update of health programmes	
3. Ensure doctors working in Attapadi visit each of the villages and interact with the community on periodic basis.	
4. Upgrade capacity of	
5. Periodic exposure and sensitisation programmes on strategies to integrate Indigenous traditions and culture into the delivery of healthcare in Attapadi	

## Data Availability

The datasets generated and/or analysed during the current study are not publicly available due to the danger of compromising the confidentiality and anonymity of the participants but are available from the corresponding author on reasonable request.

## Abbreviations

ANC: Antenatal Care
ASHA: Accredited Social Health Activist
CHC: Community Health Centre
JPHN: Junior Public Health Nurse
MMU: Mobile Medical Unit
PHC: Primary Health Centre
SDGs: Sustainable Development Goals
STDD: Scheduled Tribes Development Department
UHC: Universal Health Coverage

## Footnotes

1 Indigenous communities in India are officially referred to as Scheduled tribes. The term tribe, tribal all refer to Indigenous communities.

2 The notification of an area as a tribal block in India ensures that special attention and priority is given to the Indigenous communities living there and special developmental schemes are implemented for their well-being.
